# Challenges of implementing outsourcing of primary health services from the perspective of stakeholders

**DOI:** 10.1186/s12875-025-03045-z

**Published:** 2025-11-11

**Authors:** Hosein Ebrahimipour, Mehdi Yousefi, Saeed Tabatabaee, Elaheh Hooshmand, Ali Taghipour, Sara Jamili

**Affiliations:** 1 Department of Management Sciences and Health Economics, School of Health, Mashhad University of Medical Sciences, Mashhad, Iran; 2 Birmingham Centre for Evidence and Implementation Science, University of Birmingham, Birmingham, UK; 3 Social Determinants of Health Research Center, Mashhad University of Medical Sciences, Mashhad, Iran; 4 Department of Epidemiology and Biostatistics, School of Health, Mashhad University of Medical Sciences, Mashhad, Iran; 5 Kashmar School of Medical Sciences, Mashhad University of Medical Sciences, Mashhad, Iran

**Keywords:** Outsourcing, Primary health care, Stakeholders, Iran, Qualitative research

## Abstract

**Background:**

In recent years, outsourcing of primary health services has emerged as a strategic response to growing demands for efficiency and sustainability in healthcare systems. However, especially in low- and middle-income countries like Iran, the implementation of outsourcing faces multiple systemic and contextual challenges. This study aimed to examine the challenges associated with outsourcing primary healthcare services from the perspectives of key stakeholders in Iran.

**Methods:**

A qualitative study was conducted using directed content analysis, guided by Donabedian’s Quality of Care Model and the WHO Health System Governance Framework. Twenty-one stakeholders—including policymakers, university managers, healthcare providers, and private contractors—were selected through purposive and snowball sampling. Semi-structured interviews were conducted, and data were analyzed using MAXQDA 12 software with thematic coding until saturation was reached.

**Results:**

A total of 1,150 initial codes were extracted and grouped into 18 subthemes under four main categories: (1) Structural and policy-related challenges (e.g., lack of strong legislation, fragmented governance, contract opacity); (2) Economic challenges (e.g., low profitability, short-term contracts, economic instability); (3) Human resource challenges (e.g., job insecurity, insufficient motivation, workforce instability); and (4) Quality and service outcome challenges (e.g., declining service quality, deviation from outsourcing goals). These findings underscore the multidimensional and interdependent barriers to effective outsourcing.

**Conclusion:**

Outsourcing primary health services in Iran is hindered by a combination of legal, financial, managerial, and human resource-related challenges. To ensure effectiveness, policymakers must adopt comprehensive reforms, including national outsourcing legislation, long-term financial frameworks, transparent contract models, and performance-based management systems. Strengthening these foundational elements is critical for promoting sustainability, service quality, and private sector engagement in primary healthcare.

**Supplementary Information:**

The online version contains supplementary material available at 10.1186/s12875-025-03045-z.

## Background

The primary function of health systems is to deliver high-quality, equitable, and universal health services to populations [1]. Moreover, health systems play a crucial role in shaping national and regional economies through investments in infrastructure, employment, and service delivery [2]. In recent years, the rapid growth of healthcare expenditures has presented significant challenges to many governments in maintaining the financial sustainability of health systems [3]. As a response, structural reforms and the incorporation of private sector capacities have been increasingly considered [4]. 

One of the major policy reforms in public service delivery has been the adoption of public-private partnerships (PPP), which aim to combine the strengths of both sectors to improve efficiency, access, and service quality [5]. These changes are rooted in broader trends in New Public Management (NPM) and Public Administration Reform, which emphasize decentralization, efficiency, and market-based approaches. Underlying these reforms are theoretical models such as Social Exchange Theory [6], which highlights the importance of reciprocal relationships and trust between stakeholders, and Transaction Cost Theory, which explains the efficiency of outsourcing based on cost-benefit analysis of internal versus external service provision [7]. 

Outsourcing, also referred to as contracting-out, has emerged as a widely used tool within healthcare systems aiming to improve performance, cost-efficiency, and responsiveness. Several studies have suggested that outsourcing can increase access, equity, and service quality [8]. However, the evidence remains mixed. Some studies have highlighted that the expected cost reductions from outsourcing are often less significant than projected and that outsourcing can sometimes compromise service quality and staff satisfaction [9]. 

In countries like England, the National Health Service (NHS) has long engaged in outsourcing arrangements through PPP models, particularly for elective surgeries (e.g., cataract operations), diagnostic services, and specialized care provided by Independent Sector Treatment Centres [9]. In the United States, outsourcing of behavioral health services and managed care models is common [10]. 

In the Iranian context, outsourcing of health services has included administrative services (billing, marketing), clinical and paraclinical services, and the provision of primary health care in rural and urban areas. One notable example is the private sector’s involvement in Directly Observed Treatment (DOT) for tuberculosis control [11]. 

Nevertheless, international literature has identified major concerns regarding outsourcing in public healthcare. First, the actual financial savings are often lower than predicted [12]. Second, the delegation of service provision may weaken governmental control over essential functions, especially in clinical areas where continuity and accountability are critical [9]. 

Given the complex nature of healthcare services—characterized by human sensitivity, professional ethics, and the importance of public trust—outsourcing in this domain is fundamentally different from other sectors, Therefore, identifying the challenges and risks of outsourcing is essential for protecting service quality and equity [13]. This is particularly important in low- and middle-income countries like Iran, where systemic issues such as economic instability, weak regulatory frameworks, and workforce dissatisfaction can amplify the risks of outsourcing [14]. As the literature in this field shows, failure to address the barriers and challenges of outsourcing health services such as economic issues, responsibility and accountability, human resource challenges, structural problems and private companies, efficiency and planning challenges can lead to project failure and cause irreparable damage to the health system [15]. 

Despite previous efforts, there is still a lack of comprehensive understanding of the challenges faced during outsourcing implementation. The present qualitative study seeks to fill this gap by exploring the views of key stakeholders—including policymakers, university managers, frontline employees, and contractors—regarding the main obstacles to outsourcing primary health services in Iran.

## Method

This qualitative study employed a directed content analysis approach to explore the challenges of outsourcing primary health services to the private sector from the perspectives of stakeholders. Directed content analysis is particularly appropriate when existing theories or conceptual frameworks can guide data collection and interpretation [16]. 

In this study, the analysis was theoretically informed by two complementary models:

1. *Donabedian’s Quality of Care Model (Structure–Process–Outcome)*, which provides a systematic lens for evaluating healthcare quality through organizational infrastructure, service delivery processes, and achieved outcomes [17].
2. *The WHO Health System Governance Framework*, which highlights key dimensions for effective governance including accountability, strategic vision, stakeholder participation, transparency, and responsiveness [18, 19]. This framework was particularly useful in assessing the regulatory, financial, and contractual mechanisms involved in outsourcing [19].

These two frameworks were integrated to design the interview guide, categorize emerging codes, and interpret the implications of findings. The combination of a healthcare-specific quality model with a governance-based framework provided a comprehensive lens through which to understand the multidimensional challenges of outsourcing in the Iranian context.

### Study setting and participants

The study was conducted in 2024 in collaboration with two Medical Universities in Iran [Mashhad University of medical science – Torbat Heydariyeh University of medical science].

Participants included key stakeholders in the outsourcing of primary health services, such as university administrators, health network managers, contract specialists, staff at outsourced centers, and representatives of private contractor companies.

### Sampling and recruitment

A purposive and snowball sampling strategy was used to recruit participants who had direct experience and knowledge of the outsourcing process. Participants were contacted via Formal correspondence, telephone calls, and referrals by colleagues. Inclusion criteria included: (1) a minimum of 3 years of experience in outsourcing-related roles; (2) familiarity with operational or policy aspects of contracting-out; and (3) willingness to participate. A total of 26 individuals were approached, of whom 21 agreed to participate in the interviews. Reasons for non-participation included lack of time and unwillingness to participate. Maximum variation was considered in the selection process to capture diverse perspectives across roles, institutions, and geographical settings.

### Data collection

Semi-structured interviews were conducted using an interview guide developed based on the two theoretical frameworks. The guide was reviewed by two qualitative research experts to ensure content validity. Interview questions probed structural issues, governance constraints, human resource dynamics, and quality outcomes. All interviews were conducted by the Corresponding author, recorded with permission, and lasted between 40 and 60 min. Field notes were also taken during the interviews.

Interviews continued until data saturation was reached after 18 interviews; three additional interviews were conducted to confirm that no new themes were emerging.

### Data analysis

Data were analyzed using directed content analysis (Hsieh & Shannon) [16], with analytic steps adapted from Colaizzi’s descriptive phenomenological method to ensure interpretive depth. MAXQDA 12 software was used for coding. Codes and categories were initially mapped onto Donabedian’s model (structure–process–outcome), then contextualized using the WHO governance domains [17, 18]. To enhance trustworthiness, member checking was performed by sharing a summary of the findings with selected participants.

## Result

A total of 21 participants (11 males and 10 females) with a diverse range of responsibilities and experiences in the field of outsourced primary health services contributed to the study. The participants’ average age ranged from 23 to 52 years, with educational backgrounds from bachelor’s to doctoral levels. Participants represented three primary stakeholder groups: contractor company representatives, university-level administrators (employers), and frontline health workers (Table 1).

**Table 1:** Demographic characteristics of the participants

Variable	Category	*N* (%)
Gender	Male	11 (52.4%)
	Female	10 (47.6%)
Age	Mean Age – Male	48.67 years
	Mean Age – Female	41.23 years
Education Level	Bachelor’s Degree	12 (57.1%)
	Master’s Degree	6 (28.6%)
	Ph.D.	3 (14.3%)
Job Position	Health Network Manager	1 (4.8%)
	Health Deputy	1 (4.8%)
	Family and School Health Director	1 (4.8%)
	Disease Control Manager	1 (4.8%)
	University Financial Manager	1 (4.8%)
	Environmental and Occupational Health Expert	2 (9.5%)
	Midwife	3 (14.3%)
	Health Workers	4 (19.0%)
	Family Physician	1 (4.8%)
	Contract Officer	1 (4.8%)
	Contractor Company Representative	1 (4.8%)
	Others	4 (19.0%)

From the analysis of 1,150 open codes, 18 subthemes were identified and categorized into four main themes. (Table 2) This categorization is based on the widely-used Donabedian’s Structure–Process–Outcome (SPO) framework, which provides a robust model for evaluating the quality of healthcare. Each challenge was categorized based on its operational role and its level of influence within the framework.

In alignment with the SPO model, we categorized the challenges as follows:

- Structure Challenges: This category encompasses challenges related to the foundational resources and conditions required for outsourcing success. It includes Structural and Managerial Challenges, Economic Challenges, and Human Resources Challenges. These themes reflect issues with the organizational setup, financial resources, and the quality and stability of the workforce. all of which are fundamental structural components.
- Process Challenges: This category includes challenges related to the specific activities and procedures involved in delivering outsourced services. This includes sub-themes such as inefficient monitoring and performance evaluation systems, lack of transparency in contracts. These are the key operational elements that can compromise the quality of care.
- Outcome Challenges: This category reflects the final results of the outsourcing initiatives. It includes Service Quality and Outcome Challenges, with sub-themes such as decreasing the quality of service and moving away from the main purpose of outsourcing, which represent the ultimate consequences for stakeholders.”

**Table 2:** Main themes and sub-themes of outsourcing challenges

Main Theme	Sub Theme
Structural and managerial Challenges	Lack of Strong Supporting Legislation- Lack of unity of command- Lack of competitive environment- Customization of outsourcing- Lack of transparency in contracts- Inefficient performance monitoring system-
Economic challenges	Low profitability- Unfair valuation of services- Short-term contracts/feeling temporary- Lack of decision making authority and financial independence- Economic instability
Human Resources Challenges	Insufficient Motivational Mechanisms- Lack of job security- Workforce instability- Feeling of inequality and being overlooked -Reluctance of Specialized Medical and Dental Professionals to Work in Outsourced Healthcare Sectors
service quality and outcome challenges	Moving away from the main purpose of outsourcing- Decreasing the quality of service

### Structural and managerial challenges

#### Lack of strong supporting legislation

Because outsourcing does not have a strong protection law at the level of the legislature and the parliament, and often the laws related to outsourcing are proposed and approved at the level of the university’s board of trustees, and in the implementation, the contractor company may not be able to rely on it when faced with problems. To be supported by these laws.

From the contractor’s perspective -Participant No. 17: “*If each university implements its own law*,* we will not be able to reach a single law for outsourcing in the country. When a single law is not implemented in the country*,* legislators at high levels do not care to consider this issue as a package.” It should be considered complete*,* that is*,* for example*,* I want to implement a handover model in Torbet University. This handover model that I am doing*,* the ultimate goal is that I can approve it within the authority of the university’s board of trustees. Here*,* I can no longer go to the parliament or the ministry. I mean*,* when you enact a law that is at the level of your university’s board of trustees*,* on the other hand*,* the upper laws of the country may not be in line with your laws*,* you come up with a series of them based on what you have in mind. You take it and adapt it to the rules of the upper level. It is the same famous example that the cook who is divided into two*,* the food is either salty or unsalted. Like the family doctor who went to the level of the parliament*,* maybe we need to reach a certain form in the Ministry of Health in this handover model and go to pass as a law in the parliament some items that can be the upstream law of these contracts. Overshadow it*,* let’s amend it right there*,* for example*,* say that this law has been handed over*,* so the private sector is no longer applicable here*,* for example*,* accountable supervision is not applicable here*,* in this way*,* many interferences will be removed*.”

From the employer’s perspective **– **Participant No. 6: “*Right now*,* if the university’s board of trustees allows a 10-year contract with a private company*,* the National Audit Office intervenes and says*,* ‘Why did you sign a 10-year contract when you could’ve held ten separate tenders to potentially transfer services to the private sector at a lower cost?’ If this law is passed by Parliament*,* then the National Audit Office will no longer be able to challenge such decisions. We need strong legal frameworks to support outsourcing*.”

Human resources -Participant No.2 “*We don’t have strong legal protection. If any issue arises*,* no one stands behind us—especially when we protest about our wages or working conditions. Neither the company nor the university supports us or defends our position*.”

#### Lack of unity of command

Unity of Command, which is sometimes referred to as the chain of command, is one of the 14 principles of artistic management by Fayol, and it means that each employee must receive orders from a superior to do any work [20]. The participants in the interview stated in this regard.

From the contractor’s perspective - Participant number 12: *“Corporate personnel are recruited and selected by the university and work in the company. On the one hand*,* since the personnel is our company and receives a salary from us*,* they must be accountable to us and strive to achieve our goals. On the other hand*,* they must also be accountable to the university. Each employee has several bosses and receives orders from several bosses*,* and sometimes the orders of these bosses conflict with each other.”*

From a human resources perspective-Participant No. 8: “*We have 100 superiors*,* each of them gives an order and we have to execute it. All this is written in management books about unity of command*,* but the exact opposite of this is happening here. We have to answer to several people as leaders*.”

#### Lack of competitive environment

One of the major problems of universities during outsourcing is the lack of presence and acceptance of the private sector in this field, and practically the number of companies applying for outsourcing is very few. This has caused outsourcing companies to move towards monopoly instead of a competitive environment.

From the employer’s perspective-Participant No. 1: “*Due to the problems of outsourcing*,* applicants are very limited in outsourcing tenders*,* not having the right to choose has caused the competitive environment to disappear and on the one hand*,* instead of competition*,* we move towards monopoly.We are in a type 3 university*,* in this way*,* when we bid for outsourcing*,* we don’t have many applicants because according to our budget*,* it is not enough for the companies to work*,* that’s why the companies are limited*,* and we don’t have many applicants*,* and therefore the power We have no choice*.”

*From a human resources perspective -*Participant No.11: *We have no choice; we must work with the company currently in place. Since this is the only contracting company operating in this field*,* we have no alternatives. Whatever conditions they impose*,* we’re forced to accept them*.

#### Customization of outsourcing

The lack of precise, clear and unified rules in the field of outsourcing and assigning this matter to each university has caused outsourcing and contracting in each university to be done according to taste and there is no fixed procedure in all universities.

From the employer’s perspective -Participant No. 10: “*When there is no clear instruction to outsource services*,* our managers act arbitrarily and outsource services that could be managed with the organization’s forces without preliminary study and investigation. And instead*,* they don’t outsource some activities that private companies can do much better*.”

From the contractor’s perspective**-** Participant No.13: “*When signing a contract*,* there’s no clear or consistent process. Each university—and even each year as the board of directors’ changes—sets different regulations. For instance*,* in this term under Mr. X’s presidency*,* one service is outsourced and a new set of responsibilities and rules are imposed on the contracting company. In the next term*,* when the president changes*,* the rules may shift again based on the new president’s views. Moreover*,* outsourcing the same service might follow one set of regulations at a university*,* while in a neighboring province’s university*,* the very same service is outsourced under entirely different rules and obligation*s.”

#### Lack of transparency in contracts

Transparency in the contract means considering all possible aspects that may arise during the execution of a contract and providing a clear solution regarding these matters, but the participants in the interview stated that there are many blind spots in the current contracts that cause It does not have the necessary transparency.

From the employer’s perspective -Participant No. 5: “*Not all cases have been seen in the contracts*,* and this makes the companies not responsible in some issues that are not clear*,* for example*,* the labor department fined the company according to the labor law*,* but they put the responsibility on the university*. *Because the companies and their representatives and even those who wrote the contract in the university*,* sometimes they do not have the complete authority to perform those services and they have not had executive work*,* they do not include a series of issues in the contracts*,* this causes many problems later*,* because the first thought We haven’t done it*,* now when we deal with the problem*,* we get confused*,* but if the professional forces who have done the field work are present during the contracts*,* the contracts will be more transparent and many of these problems will not occur anymore*.”

From the contractor’s perspective- Participant No.18: *The contract clauses are open to interpretation. When the university drafts the contract*,* the provisions are written in a vague and generalized manner. So*,* when challenges arise*,* the university offers different interpretations to avoid legal responsibility and shifts the meaning in its favor*.

#### Inefficient performance monitoring system

One of the key challenges faced by universities in outsourcing is the absence of a robust performance monitoring and evaluation system. Without reliable verification mechanisms, it becomes difficult to accurately assess the performance of private sector providers.

From the employer’s perspective -Participant 9: *“Performance evaluation refers to the process of assessing the success or failure of staff activities based on predetermined performance indicators. In the absence of strong metrics*,* it’s impossible to make a fair judgment about success or failure.”*

*From the contractor’s perspective-* **-**Participant NO.3:*“The performance evaluation indicators that have been defined are imprecise and influenced by personal preferences.”*

### Economic challenges

#### Low profitability

The next challenge was the low profitability. Due to the low pricing of outsourcing contracts, the amount paid to the company—after deducting expenses and paying staff salaries—leaves only a small sum as managerial profit for the companies. Compared to the challenges and complications associated with outsourcing, this amount is extremely limited and has significantly reduced the incentive for private sector participation in outsourcing initiatives.

From the employer’s perspective -Participant No. 15: “*"With the limited resources allocated for outsourcing*,* our budget is quite restricted. The payment made to companies through outsourcing contracts yields little profit for them. As a result*,* our bargaining power is significantly weakened*,* reducing our influence in outsourcing arrangements.”*

From the contractor’s perspective- Participant No.18: " *It has a small profit that is not worth the trouble at all*,* which is why no company or private sector wants to enter this field. The health sector is not very attractive for the private sector because of its nature*,* it is necessary to provide free services to the people and you cannot earn money in this way*.”

#### Unfair valuation of services

During the implementation of service purchasing and pay-for-performance schemes, one of the most critical issues to address is the valuation of services. Through this process, we can achieve greater transparency in services and their value, paving the way for proper outsourcing. However, participants indicated that, at present, service valuation is being conducted unfairly.”

From the contractor’s perspective Participant No. 12: “*In the beginning*,* when the volume and definition of the health sector services were done*,* this volume was correct*,* but what was wrong in the middle was the valuation of the services. The valuation of the services was based on savings. It was a manpower. Look*,* if we see everything in reality that is implemented everywhere and is not comparable*,* it is great. The working hours are per day. The performance-based payment system means that if you want to get the salary equal to 8 hours per day*,* you must also do the secretary’s work in addition to being the secretary of the boss.*

From a human resources perspective -Participant No. 14 : *“The tariffs set for services in the healthcare sector are not realistic. Different services vary in terms of workload and difficulty*,* yet they’re assigned equal tariffs and valued uniformly. As a result*,* we end up working much more than what we’re compensated for.”.*

#### Short-term contracts - feeling temporary

Pursuant to the directives issued by the Ministry of Health and the National Inspection Organization, outsourcing contracts in the healthcare sector must be limited to a one-year term. This policy is intended to facilitate annual bidding processes, thereby allowing capable companies offering competitive pricing to participate. However, this short-term contractual framework has inadvertently fostered a perception of instability among service providers. Contractors frequently express concerns regarding the temporary nature of their engagement, which constrains their strategic planning and discourages long-term investment.

From the contractor’s perspective-Participant 7: “*A series of items should be amended. For example*,* the requirement that all contracts must be signed for one year. This is a law that is not known for what purpose it was signed and what was the background of this law. Good. Obviously*,* no private company with any vision is willing to sign a one-year contract. As a private company*,* I put in the best performance and best energy to perform well*,* if a company comes next year and gives a lower price and wins the tender*,* what is my duty? I have to be unemployed*.”

From a human resources perspective -Participant No. 11: “*The companies sign one-year contracts with the university*,* so the contracts issued to us are also for one year. Each year*,* we live with the fear that the company may not return the next year*,* and we could end up unemployed”*.

#### Lack of decision-making authority and financial independence

One of the most significant challenges in outsourcing initiatives has been the lack of decision-making authority and financial autonomy. This constraint has hindered the alignment of current outsourcing practices with the fundamental principles of effective delegation. In this regard, several participants noted:

From the employer’s perspective -Participant No. 10 : “*Outsourcing has been done*,* but in practice*,* we did not proceed according to the main principles of outsourcing. The first principle in outsourcing is to give financial independence to the private sector*,* that is*,* in exchange for the money we give you*,* we want you to manage a special center for us. We want this quality from you*,* we will pay this much*,* and you don’t have to deal with the details anymore*.”

From the contractor’s perspective-Participant No. 13: “*When the company does not have the right to make decisions and practical independence in any field*,* it cannot have management*.”

#### Economic instability

Economic instability in the country—including severe currency fluctuations, continuously rising costs, and the absence of a stable financial outlook—is a key factor undermining the effective implementation of healthcare service outsourcing. Such instability disrupts the efficacy of contracts, budgets, and medium- to long-term planning processes. The literature highlights that economic volatility poses a serious threat to the sustainability and quality of health services delivered by the non-governmental sector [21]. 

From the contractor’s perspective-Participant No. 3: “*When a company comes and wins the tender and starts working*,* it is the first problem*,* because with this economic situation*,* prices change every day*,* but the company receives the same money as written in the contract in the first year. Many times*,* companies They face problems in supplying their equipment and consumables*,* which means that at the end of the year*,* with these prices*,* the company has not made a profit and has nothing left*.”

From the contractor’s perspective:- Participant No.18: “*Due to unstable economic conditions and the continuous rise in prices*,* our budget constraints have become a serious obstacle to signing new contracts with private contractors. The outsourcing payments we propose are deemed unacceptable*,* as they fail to align with the contractors’ current operational costs. Moreover*,* sudden and significant price fluctuations disrupt the contractors’ cost management*,* potentially leading to a decline in the quality and quantity of services delivered. As a result*,* we are frequently compelled to amend contracts and raise the allocated budget in order to maintain service quality.*”

### Human resources challenges

#### Insufficient motivational mechanisms

Motivation is an internal driving force that determines the intensity, direction, and persistence of an individual’s effort toward achieving a goal [22]. In other words, it is an inner state that generates energy and defines the direction and magnitude of behavior aimed at fulfilling a need [23]. Effective organizational management requires that managers, by understanding and anticipating the motivations of their workforce, actively strive to meet their needs appropriately and timely—thereby creating the conditions for genuine engagement and productivity aligned with organizational goals [24]. In this regard, participants stated:”

From the contractor’s perspective-Participant No. 3: “*The forces are completely unmotivated. The company oppresses them a lot. Of course*,* these are educated forces*,* they know their rights better*,* but the law does not help them much. They usually compare themselves with other forces when they are so They see discrimination and difference*,* they become unmotivated and it affects their work*.

From a human resources perspective- Participant No. 2 : “*For contracted personnel*,* there are no specific reward items defined. Beyond our basic salary*,* we receive no additional benefits. As a result*,* we have no motivation to work harder or perform better. In fact*,* we have no incentive to remain in this position at all.”*

#### Lack of job security

Perceived job security is defined as the sense of safety regarding the preservation and continuity of one’s employment. It is considered one of the key manifestations of feeling secure and, following physiological needs, constitutes the strongest level of human motivation [25]. In the workplace, this need is fulfilled by assurance of continued employment [26]. In essence, perceived job security reflects an individual’s assessment of personal, organizational, and environmental conditions, leading them to the conclusion that no particular factor threatens their job stability—and that they can confidently rely on continued employment both now and in the future. If organizations fail to incorporate modern concepts of job security, they will not be able to achieve adequate efficiency and effectiveness in employee performance. Job insecurity undermines employee empowerment across specialized competencies, practical initiative, experiential learning, job satisfaction, work ethics, and promotion opportunities. As a result, organizations are unable to meet their specialized and social expectations [27]. In this regard, several participants stated:”

From the contractor’s perspective-Participant No. 4: “*The short duration of university outsourcing contracts*,* which are mostly one year*,* and on the other hand*,* the difficult conditions and the contract renewal process with contractors have caused most contractors to work in an environment where both they and their personnel do not feel job security. We don’t know how long to write a contract with our employee and we are losing most of our expert staff because of this*.”

From a human resources perspective -Participant No. 16: “*Every day we have to be stressed that the conditions of the decisions may change or a new company may come and they don’t want us anymore*.”

#### Workforce instability

One of the key challenges in the implementation of outsourcing in primary healthcare services is the high rate of staff turnover and attrition within contractor-managed facilities. Human resource instability can lead to diminished service quality, reduced public trust, and increased training and management costs for both contractors and commissioning authorities.

International studies have also highlighted that workforce instability—particularly within outsourced structures—results in job dissatisfaction, weakened continuity of care, and inefficiencies in healthcare delivery [28]. In the conducted interviews, this challenge was discussed from multiple perspectives:”

From the employer’s perspective -Participant No. 19: “*The university spends money*,* conducts tests*,* recruits*,* trains*,* spends and delivers the company*,* and when the contract of the company ends*,* these forces remain undecided. New forces come again and this process is repeated again*.”

From a human resources perspective - Participant No. 8 :*When there’s no guarantee for job continuity*,* it’s only natural to move on if a better opportunity comes along. This job lacks stability*.

#### Feeling of inequality and being overlooked

According to Adams’ “equality” theory, job satisfaction is the result of the behavior that is done towards us compared to others. Members and employees of the organization do not work in a vacuum. They always compare themselves with “others” [29]. The basis of comparison (other) may be a member of the same work group, or another person in other departments of the organization, or even a group of people outside the organization. After evaluating how the organization deals with “person” and “other”, the results of the evaluations are compared with each other and the person compares his position with another position. The result of this comparison for a person may be a feeling of equality or inequality. Adams describes the process of comparing equality in terms of input and output ratios. Data means what the individual gives to the organization, such as education, experience, effort, and loyalty. Staff also received the person from the organization in return, such as salary, reputation, social relations and internal rewards. One part of the evaluation of the data and data is based on objective observations (for example, the rights of the individual) and the other part is based on the perception of the individual. The following relationship represents equality comparison.

A person compares his data-to-data ratio with another data-to-data ratio. In the theory of equality, it is said that employees and members of the organization, if they feel unfair towards themselves, may reduce the level of their activity or distort or disrupt the level of consumption of themselves and others (as well as efficiency). They behave in such a way that others are encouraged to work less, reduce their consumption (data) and productivity, change the reference or what the person compares himself with, or leave the organization [30]. This issue was raised as one of the challenges of the workforce and in this regard the participants had the title.

From a human resources perspective-Participant No. 20: “*Official employees of the same rank and our colleagues have less workload and more income than us*,* We do the same work in the same room*,* we have the same degree*,* but because we are a corporate force and they are official*,* our salaries and benefits differ from earth to heaven*.”

From a human resources perspective-Participant No. 11: “*When working*,* they tell us that you are also an employee of the same system and you have to work for the system*,* but when it comes to bonuses and other benefits*,* it’s as if we don’t exist at all*,* they don’t see us at all*.”

#### Reluctance of specialized medical and dental professionals to work in outsourced healthcare sectors

In addition to challenges arising from the limited engagement of private sector applicants in outsourcing initiatives, the recruitment of specialized personnel—particularly physicians and dentists—in underserved and remote regions poses a significant challenge for university-affiliated health services, due to the low attractiveness of such positions.

From the employer’s perspective-Participant No. 3: *“The payment amount specified in the instruction (executive instruction for the provision and promotion of primary health care in urban and rural areas) is very low. As a result*,* general practitioners and dentists show no interest in joining. This makes it extremely difficult for us to recruit doctors for these regions. Those who do come don’t stay long because the working conditions and pay are unstable.”*

### Service quality and outcome challenges

#### Moving away from the main purpose of outsourcing

The involvement of the university in outsourcing challenges and affairs has caused outsourcing to move away from its main path and main goal.

From the employer’s perspective -Participant No. 19: “*We are so worried about cost*,* recruitment and tendering that we forget that the purpose of outsourcing is to improve the quality and efficiency and reduce the size of the government. If we consider these goals*,* our work will go much better. It may take more time and patience*,* but in the end*,* we will get a good result*,* but unfortunately*,* in the discussion of outsourcing that is being done now*,* it seems that the main goal of outsourcing has been completely forgotten*.”

From the contractor’s perspective **-** Participant No.4: “*The university’s current focus in outsourcing is solely on costs and the quantity of services. The emphasis on expenses is so strong that quality is essentially overlooked.”*

#### Decreasing the quality of service

*One of the significant and concerning consequences of outsourcing healthcare services is the decline in the quality of services provided to the community. Although outsourcing begins with the goal of increasing efficiency*,* expanding service coverage*,* and improving quality*,* in practice*,* the lack of effective supervision*,* unstable financial incentives*,* workforce instability*,* and resource limitations may lead to a noticeable decrease in the quality of care.*

Numerous studies have shown that without careful contract design, weak oversight, and financial pressures, outsourcing can lead to diminished professional standards and lower client satisfaction [8]. In this regard, the participants stated that:

From the employer’s perspective -Participant No.1: “*Company forces that have a temporary status are not motivated to work*,* and sometimes they are only satisfied with the completion of programs and move more towards paperwork than they want to have real performance*.”

From the contractor’s perspective Participant No.12: “*We try to maintain quality*,* but when resources are limited and the staff is unmotivated and constantly changing*,* it’s difficult to sustain it*.”

## Discussion

This study explores the challenges of outsourcing primary healthcare services in Iran from the perspective of key stakeholders and identifies four main categories of challenges: structural and policy-related, economic, human resource, and quality and service outcomes. While aligning with existing literature on healthcare outsourcing, the findings offer unique insights within the Iranian context.

### Structural and managerial challenges

The findings highlight the necessity of robust, integrated national support regulations. As noted by Niknam et al. [21] the absence of comprehensive and stable guidelines is a major obstacle. Lack of unified command and fragmented directives between the employer (university) and contractor significantly impacts operational efficiency and staff morale. This reveals a deficiency in a transparent and well-defined participatory framework, which is implicitly referenced in studies such as the Moheb-Yas Hospital case [31]. The absence of competitive environments and contractual transparency leads to monopolistic tendencies and legal ambiguities—issues also addressed in Niknam et al. [21] and in the Isfahan case study [32], both highlighting complex bureaucratic barriers and the lack of precise contracts. Furthermore, weaknesses in oversight systems hinder accurate performance evaluation and assurance of outsourcing goals.

### Economic challenges

Low profitability and unfair service valuation discourage private sector participation. These findings align with a scoping review on Challenges and Solutions in Outsourcing Healthcare Units [33], which identifies payment and management reform as critical issues. The short-term nature of contracts (often one year) creates instability for contractors, exacerbates job insecurity among staff, and undermines strategic investment—consistent with findings from the Moheb-Yas Hospital study [31] on the lack of long-term budgeting. The absence of decision-making authority and financial autonomy further limits contractors’ ability to manage services effectively [21]. Additionally, economic instability and currency exchange fluctuations significantly complicate financial planning and contract execution, which is consistent with the findings of the study on the impact of economic instability on public health spending [34] and Niknam et al. [21]. on “economic instability”.

### Human resource challenges

Insufficient motivational mechanisms, a sense of discrimination, and job insecurity emerge as major concerns. These findings strongly align with studies such as Saharkhiz et al. [35], which discuss “notable discrimination,” and Hatami et al. [36], which emphasize “inequity in payments.” Such disparities and perceptions of injustice diminish staff loyalty, leading to high workforce turnover and service instability. Research shows that job insecurity reduces employee engagement [37]. Moreover, reluctance among medical and dental professionals to work in outsourced units in deprived areas stems primarily from inadequate compensation and lack of sufficient incentives.

### Service quality and outcomes challenges

The study indicates that the university’s emphasis on cost-saving and recruitment often diverges from outsourcing’s primary goals of enhancing quality and efficiency. This echoes Niknam et al. [21] findings regarding the “lack of clear outsourcing objectives and deviation from the core mission.” Weak supervision, unstable workforce, and absence of sustainable incentives collectively diminish service quality. As Liu et al. [8] noted, without careful contract design, poor oversight and financial constraints can erode professional standards and compromise customer satisfaction.

### Strengths and limitations

This study possesses several key strengths that contribute to the credibility and depth of its findings. Firstly, by employing a comprehensive qualitative approach utilizing directed content analysis, it provided profound insights into the challenges of outsourcing primary healthcare services in Iran. A robust theoretical framework, encompassing Donabedian’s Quality of Care Model (Structure-Process-Outcome) and the WHO Health System Governance Framework, was utilized, allowing for a multi-dimensional and precise analysis of the challenges. Furthermore, a diverse range of key stakeholders, including policymakers, university managers, healthcare providers, and private contractors, were purposively and snowball-sampled. Semi-structured interviews were conducted with 21 participants until data saturation was achieved, ensuring the richness and depth of the findings.

However, this study also has several limitations. Primarily, its focus on the Iranian context restricts the direct generalizability of the findings to other healthcare systems or countries. The qualitative nature of this research also means that the findings are not statistically generalizable to a broader population, as the main objective is to provide in-depth, contextual insights. Moreover, due to the self-reported nature of the interview data, there is a potential for social desirability bias in participants’ responses. Finally, reliance on self-reported data might, in some instances, affect the comprehensiveness of understanding certain phenomena, although efforts were made to enhance the trustworthiness of the findings through methods such as member checking and peer debriefing.

## Conclusion

This study clearly demonstrates that, despite its theoretical potential, outsourcing primary healthcare services in Iran faces significant structural, economic, human resource, and quality-related challenges in practice. The central finding underscores that unless these challenges are comprehensively addressed, key outsourcing goals—namely improving efficiency, reducing costs, and enhancing service quality—cannot be achieved. A short-term cost-saving focus, without considering long-term consequences for quality and human resources, proves ineffective. By offering an in-depth analysis of stakeholder perspectives, this research contributes to a more comprehensive understanding of existing barriers in Iran’s primary healthcare system and lays the groundwork for future reforms. Such reforms may include enacting a national outsourcing law with enforceable mandates, designing medium-term contracts with long-term financial and legal guarantees, and establishing transparent, precise systems for performance evaluation and human resource motivation management. These changes could foster sustainable and productive private sector participation and represent a meaningful step toward realizing equity and quality in health service delivery.

## Supplementary Information

Supplementary Material 1.

## Data Availability

The data that supports the findings of this study is the result of interviews with people who have opinions about the subject under study, which was developed only for the purpose of this study. This data is not publicly available due to privacy or ethical restrictions. And only one example of this interview is available in the supplementary files section. The rest of the interview data is available upon request from the corresponding author.
