# Supplementary material for: Challenges of implementing outsourcing of primary health services from the perspective of stakeholders
Source: BMC Prim Care. 2025 Nov 11;26:355. doi: 10.1186/s12875-025-03045-z (PMC12606836; doi:10.1186/s12875-025-03045-z)
Supplement: Supplementary file 1 — Supplementary Material 1. [file 12875_2025_3045_MOESM1_ESM.docx]

# COREQ Checklist – Consolidated Criteria for Reporting Qualitative Research

This checklist includes 32 items grouped into three domains. It has been completed based on the qualitative study entitled 'Challenges of Implementing Outsourcing of Primary Health Services from The Perspective of Stakeholders'.

| Domain / Area | Item Description | Response in this Study |
| --- | --- | --- |
| Personal Characteristics | Interviewer/facilitator | The interviews were conducted by the Corresponding author. |
|  | Credentials | The Corresponding author holds a PhD in Health Services Management. |
|  | Occupation | University faculty member in health management. |
|  | Gender | Female |
|  | Experience and training | Experienced in qualitative research and content analysis training. |
| Relationship with participants | Relationship established | Some participants had previous professional relationships with the researcher. |
|  | Participant knowledge of the interviewer | Participants were informed about the research goals and interviewer background. |
|  | Interviewer characteristics | The interviewer maintained neutrality and had relevant qualitative experience. |
| Theoretical framework | Methodological orientation and theory | Directed content analysis using Donabedian Model and WHO governance framework. |
| Participant selection | Sampling | Purposive and snowball sampling to ensure maximum variation. |
|  | Method of approach | Participants were contacted via Formal correspondence, phone and professional networks. |
|  | Sample size | 21 participants (managers, staff, contractors). |
|  | Non-participation | 5 individuals declined due to time constraints or disinterest. |
| Setting | Setting of data collection | Offices or via phone due to scheduling constraints. |
|  | Presence of non-participants | No. |
|  | Description of sample | Diverse in gender, job level, and institution; summarized in Table 1. |
| Data collection | Interview guide | Developed based on theory; pilot tested. |
|  | Repeat interviews | No. |
|  | Audio/visual recording | All interviews were audio recorded. |
|  | Field notes | Yes, taken during interviews. |
|  | Duration | Each interview lasted 40–60 minutes. |
|  | Data saturation | Achieved after 18 interviews; 3 additional for confirmation. |
|  | Transcripts returned | Summaries were shared with participants for verification. |
| Data analysis | Number of data coders | Two: the first author and a research assistant. |
|  | Description of coding tree | Explained in method; conceptual map developed. |
|  | Derivation of themes | Themes derived from theoretical framework and data. |
|  | Software | MAXQDA 12 |
|  | Participant checking | Findings validated by participants. |
| Reporting | Quotations presented | Yes, with participant role and number. |
|  | Data and findings consistent | Yes, supported by direct quotes. |
|  | Clarity of major themes | Yes, four main themes clearly reported. |
|  | Clarity of minor themes | Yes, 18 subthemes are clearly detailed. |
